# Supplementary material for: Swelling-strengthening hydrogels by embedding with deformable nanobarriers
Source: Nat Commun. 2020 Sep 9;11:4502. doi: 10.1038/s41467-020-18308-9 (PMC7481780; doi:10.1038/s41467-020-18308-9)
Supplement: Supplementary file 1 — Supplementary Information [file 41467_2020_18308_MOESM1_ESM.pdf]

Supplementary Information

Swelling-strengthening hydrogels by embedding with deformable nanobarriers

Wu et al.

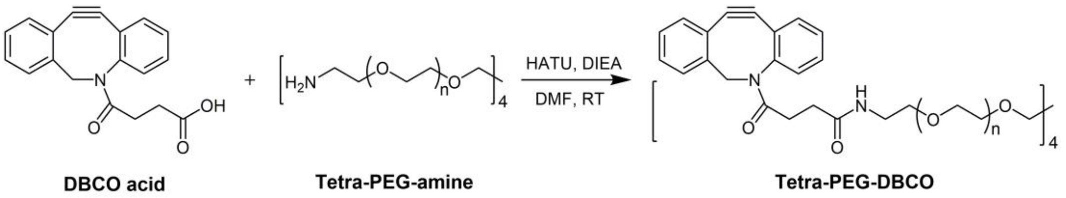

Supplementary Figure 1. Synthetic route of Tetra-PEG-DBCO.

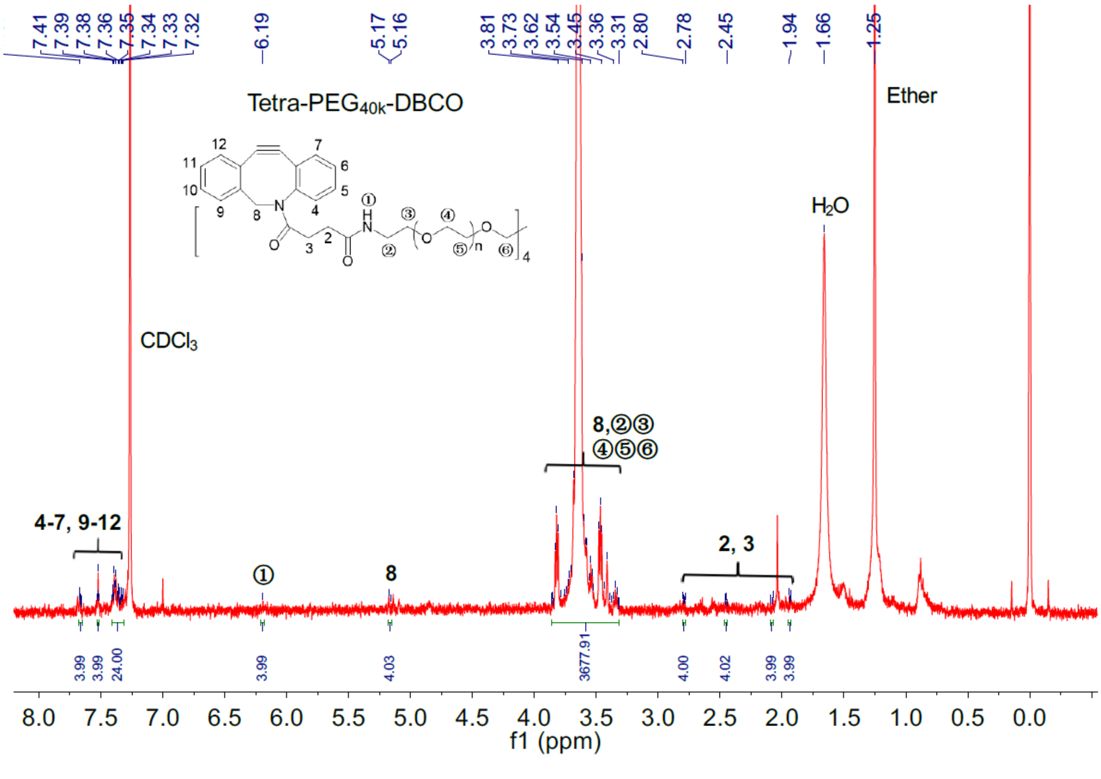

Supplementary Figure 2. ^{1}H NMR spectrum of Tetra-PEG-DBCO.

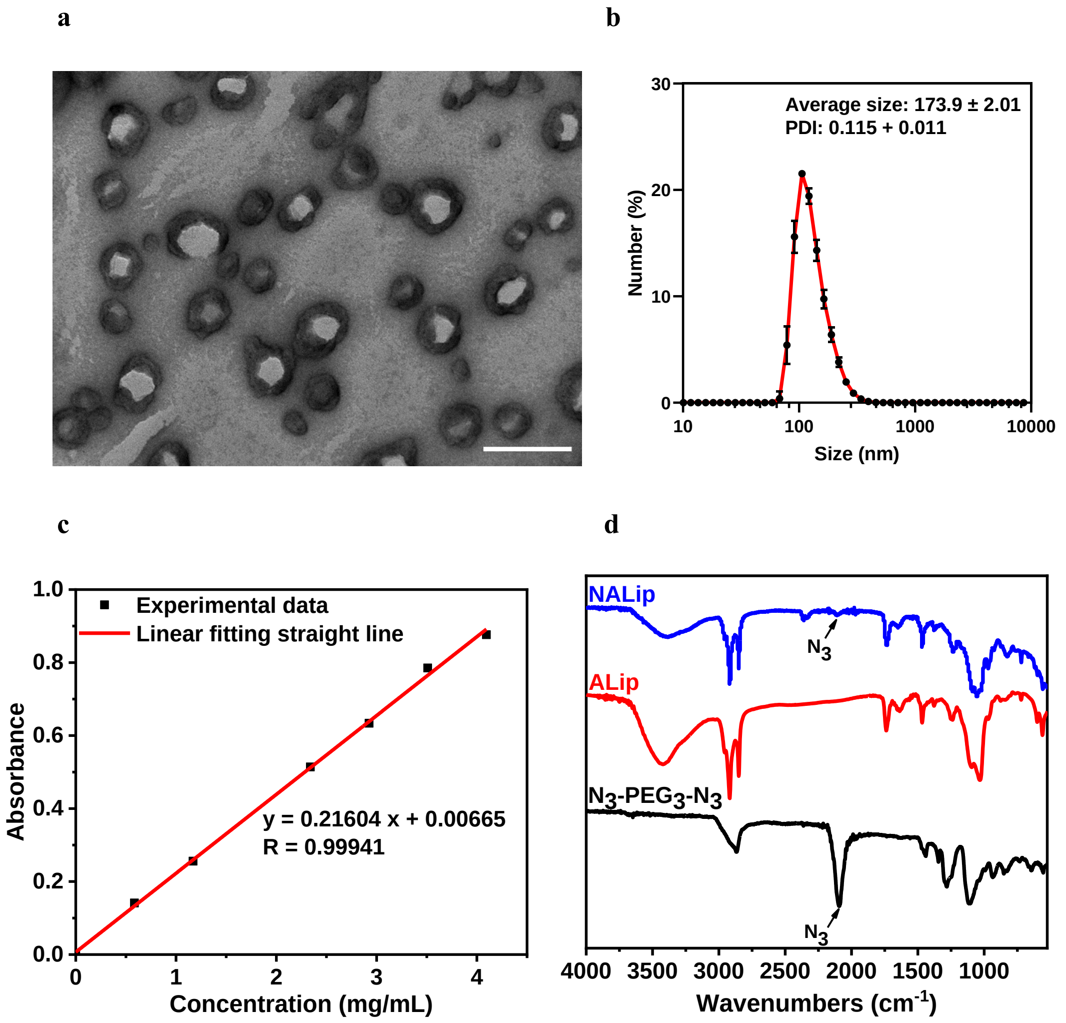

Supplementary Figure 3. a Negatively stained TEM image and **b** size distribution of ALip. Scale bar, 200 nm. Error bars represent the mean ± SD (*n* = 3 independent experiments). **c** Standard curve of absorbance versus concentration of N_3-PEG_3-N_3. **d** FTIR spectra of N_3-PEG_3-N_3, lyophilized ALip and NALip.

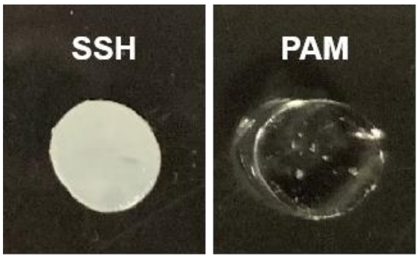

Supplementary Figure 4. Photographs of SSH and PAM hydrogels.

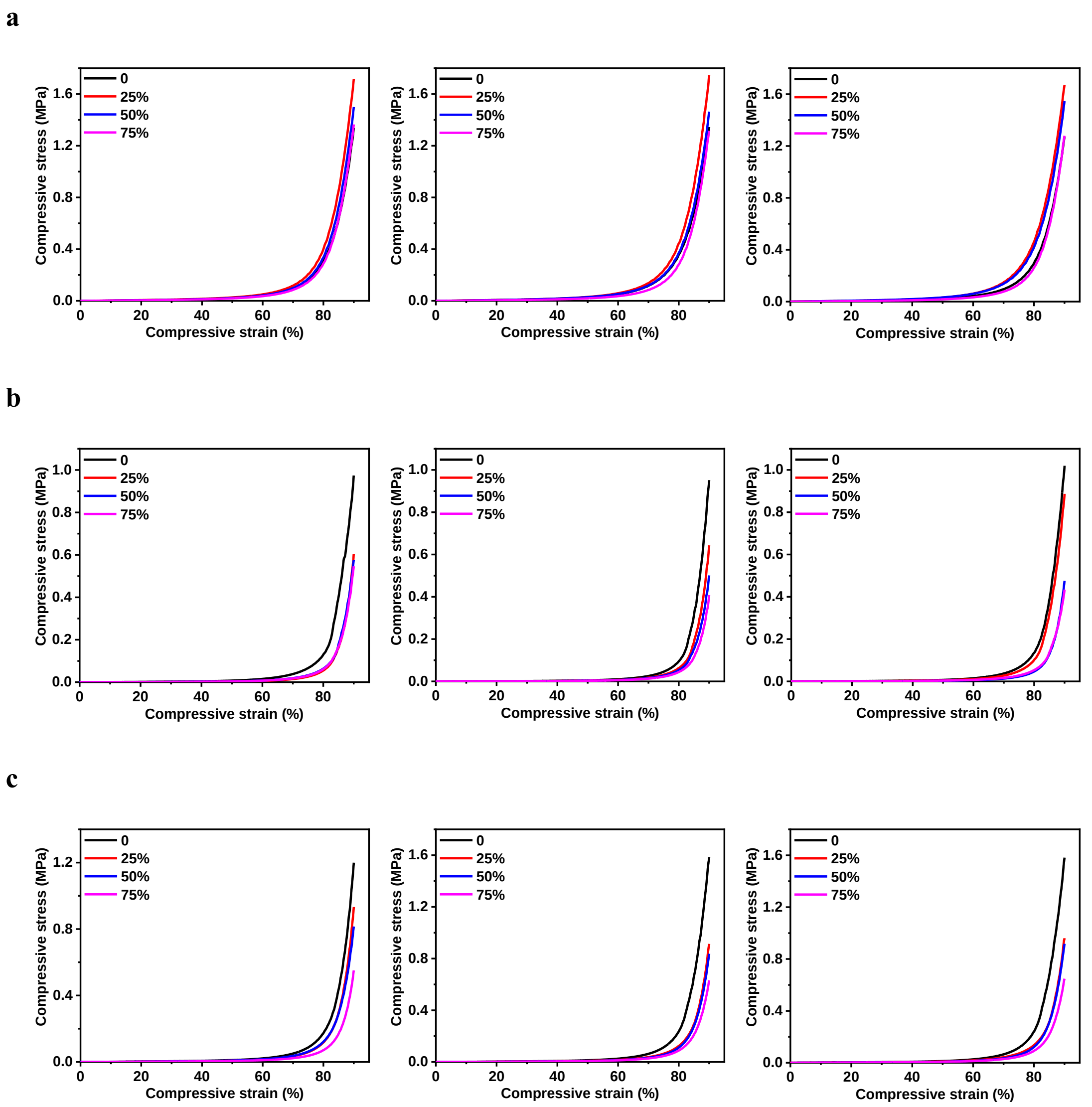

Supplementary Figure 5. Compressive stress versus strain curves of **a** SSH, **b** PAM and **c** PAM/PEG hydrogels under different swelling degrees at a strain of 90%.

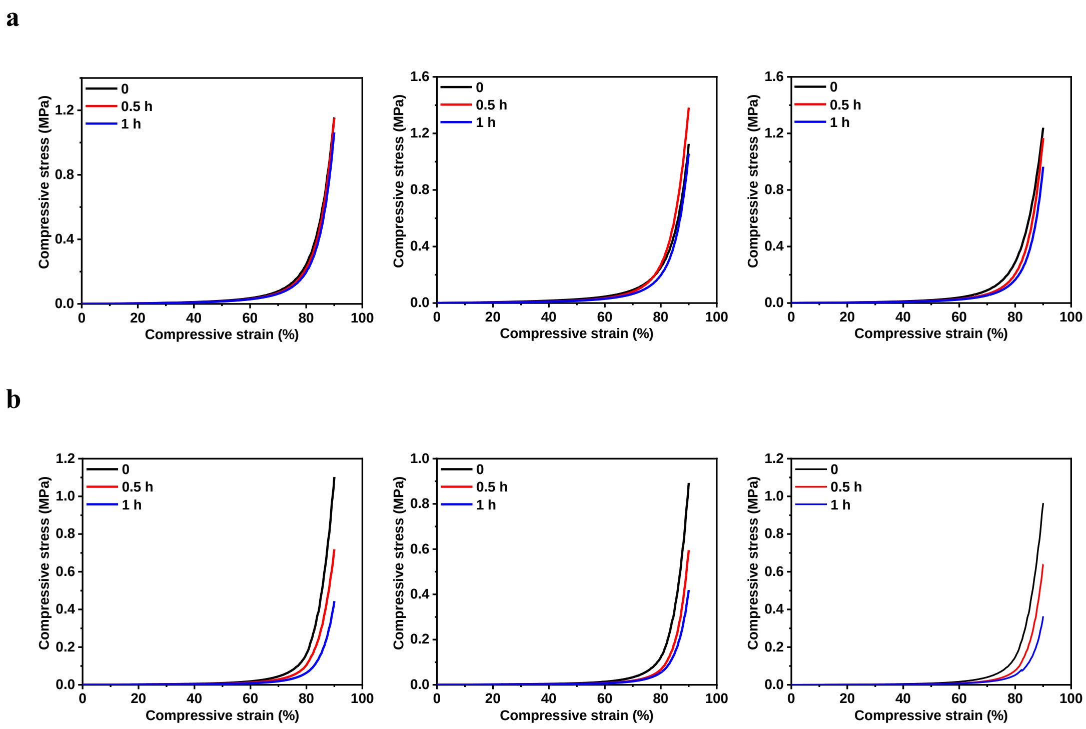

Supplementary Figure 6. Compressive stress versus strain curves of **a** SSH and **b** PAM hydrogels after different implantation time at a strain of 90%.

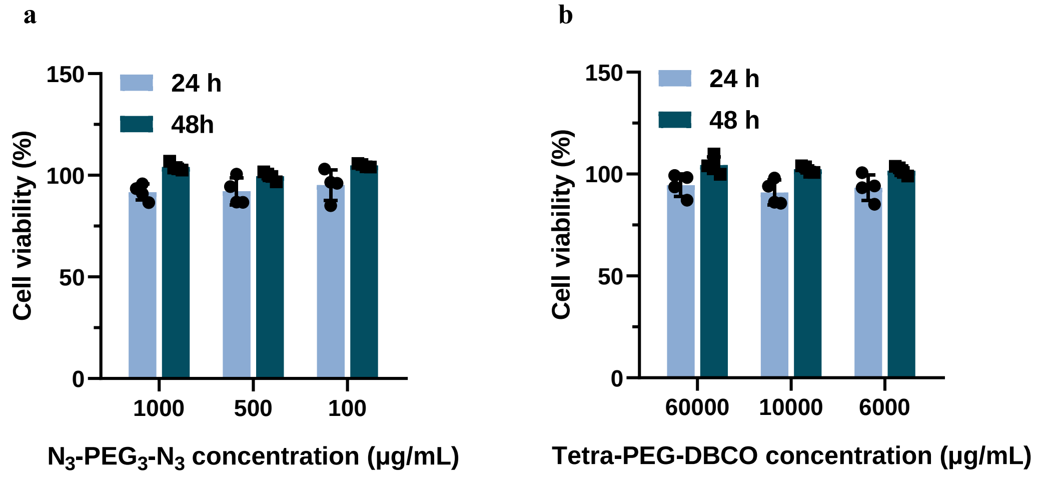

Supplementary Figure 7. Cell viability analyses of **a** N_3-PEG$_3$-N_3 and **b** Tetra-PEG-DBCO by CCK-8 assays. Cells were incubated for 24 and 48 hours respectively and the viability was monitored by measuring the values at OD450. Error bars represent the mean ± SD (n = 4 independent experiments).

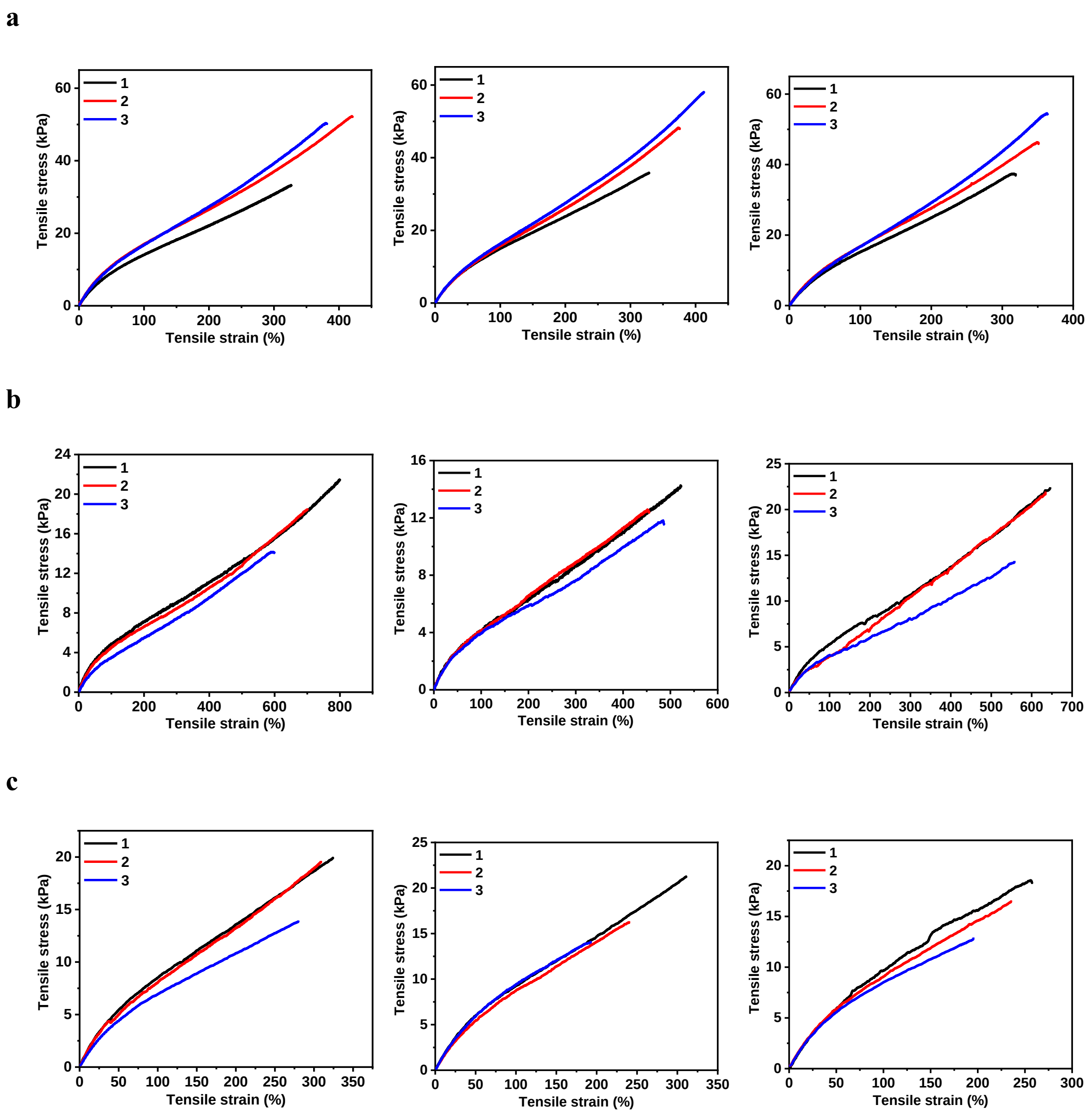

Supplementary Figure 8. Tensile stress versus strain curves of **a** SSH, **b** PAM and **c** PAM/PEG hydrogels under different tensile conditions. 1: directly test; 2: test after prestretching at 100% strain for 5 min; 3: test after prestretching at 200% strain for 5 min.

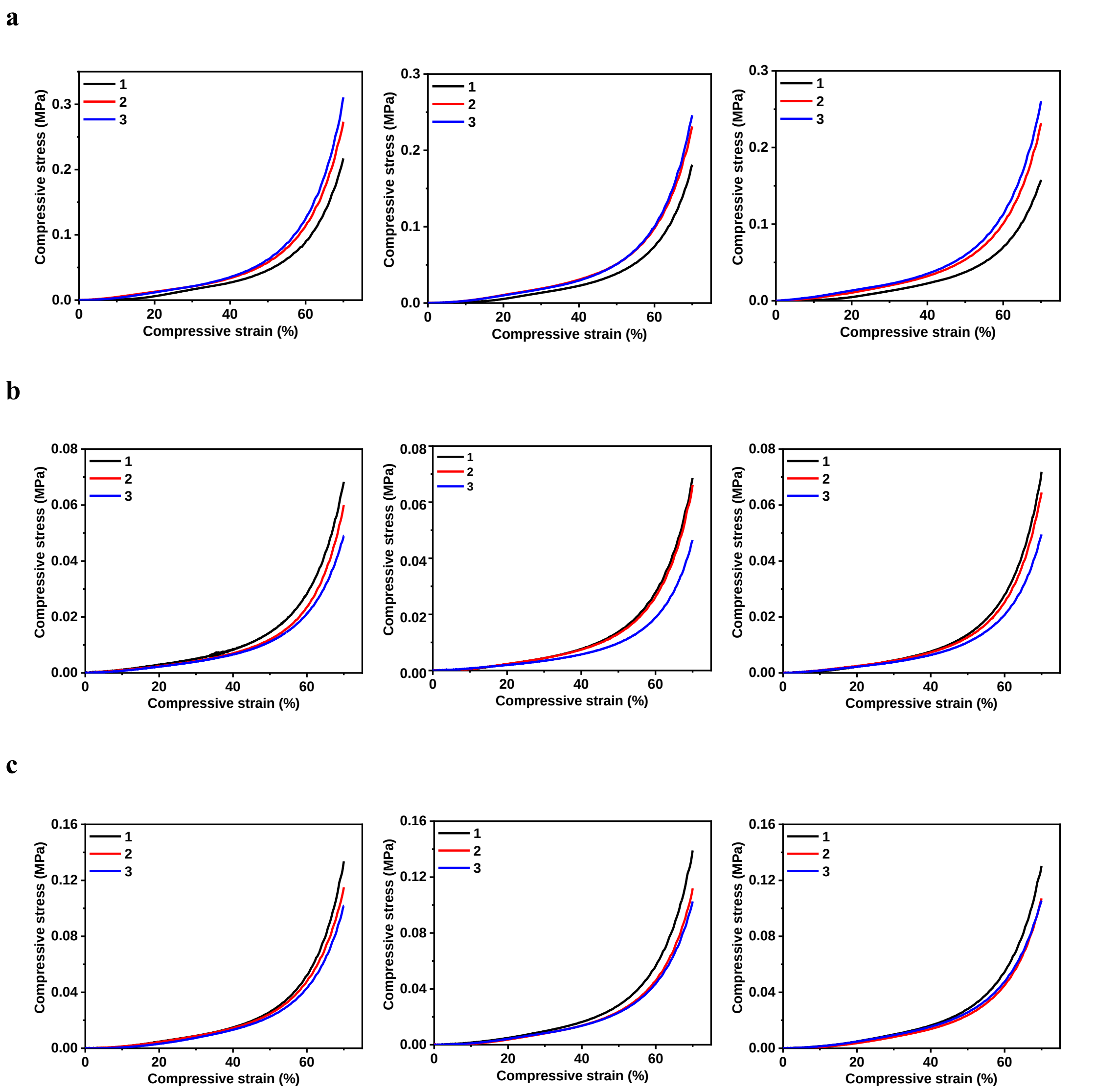

Supplementary Figure 9. Compressive stress versus strain curves of **a** SSH, **b** PAM and **c** PAM/PEG hydrogels under different compression conditions. 1: directly test; 2: test after compression at 70% strain for 5 min; 3: test after compression at 70% strain for 10 min.

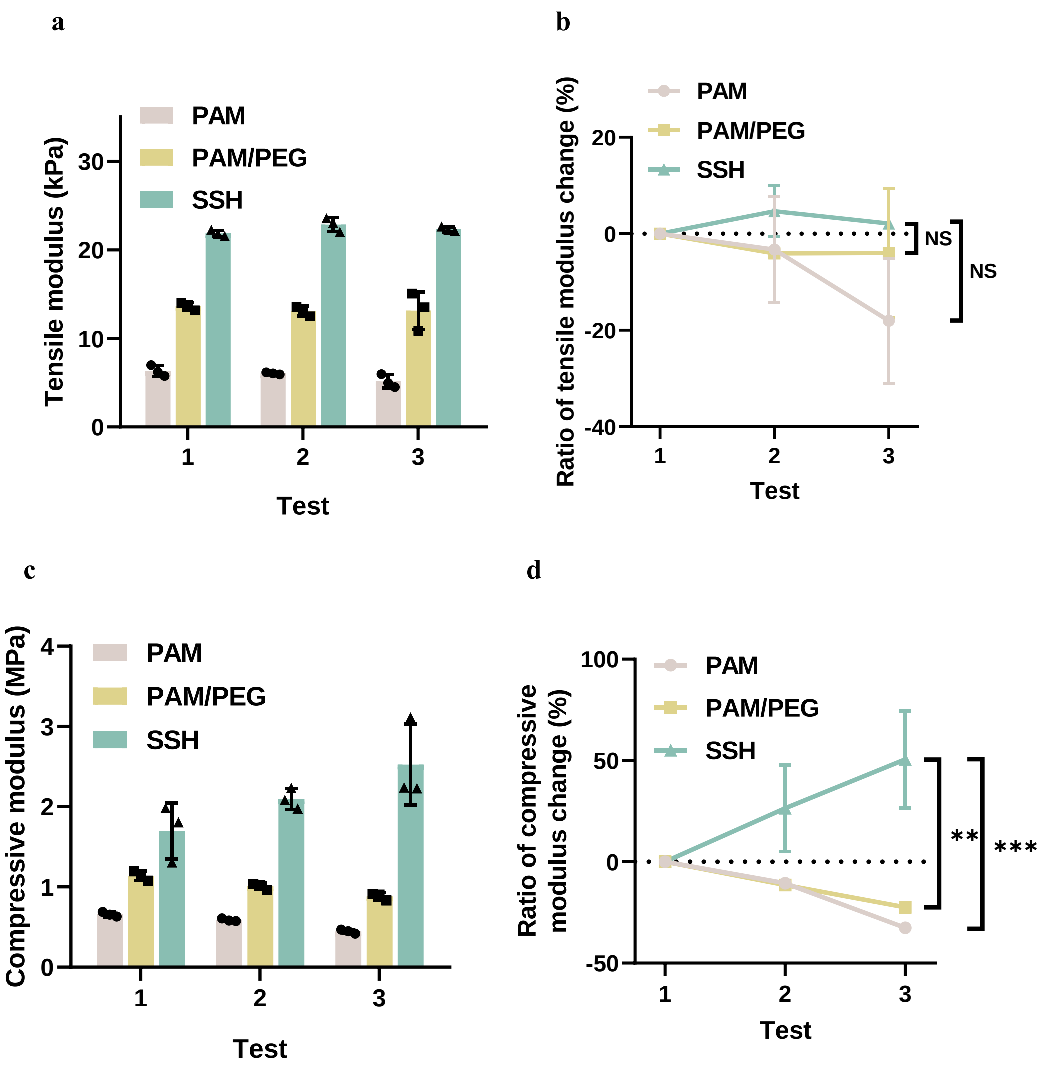

Supplementary Figure 10. **a** Tensile modulus and **b** variation of tensile modulus under different tensile conditions. 1: directly test; 2: test after prestretching at 100% strain for 5 min; 3: test after prestretching at 200% strain for 5 min. **c** Compressive modulus and **d** variation of compressive modulus under different compression conditions. 1: directly test; 2: test after compression at 70% strain for 5 min; 3: test after compression at 70% strain for 10 min. All error bars represent the mean ± SD (n = 3 independent experiments). Significance was assessed using one-way ANOVA with Tukey's multiple comparisons test, giving p-values, $**p < 0.01$, $***p < 0.001$. NS: no significance.

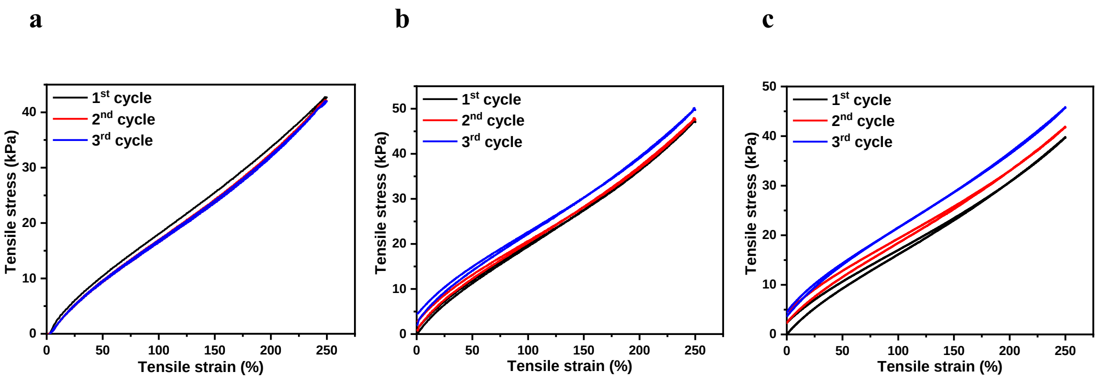

Supplementary Figure 11. Cyclic tensile tests. SSH samples were stretched to 250% for 3 cycles at different tensile rates. **a** 200 mm/min, **b** 50 mm/min and **c** 5 mm/min.

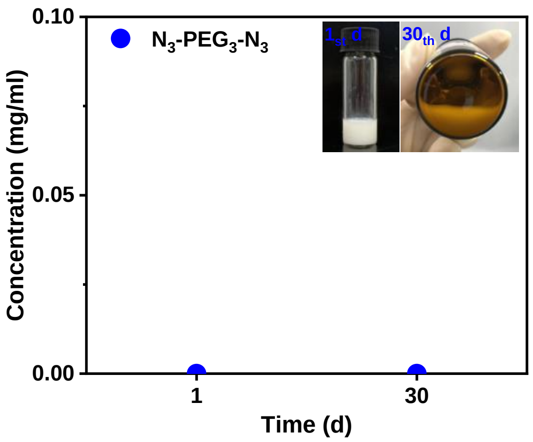

Supplementary Figure 12. Concentration of N_3-PEG_3-N_3 in the subnatant of NALip solution after different storage time. The concentrations of the released crosslinker were set at zero as no significant signals could be observed during this period. Inserts represent the photographs of the corresponding liposomal solution.

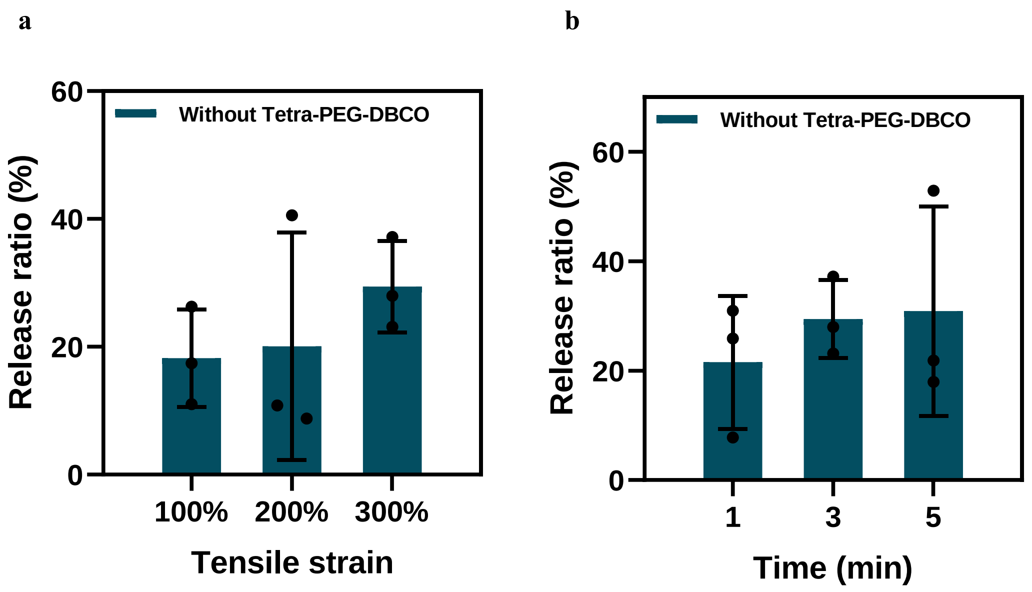

Supplementary Figure 13. Release ratio of N_3-PEG_3-N_3 in SSH under different tensile conditions. **a** Stretching at different strain for 3 min. **b** Stretching at 300% strain for different time points. All error bars represent the mean ± SD (*n* = 3 independent experiments).

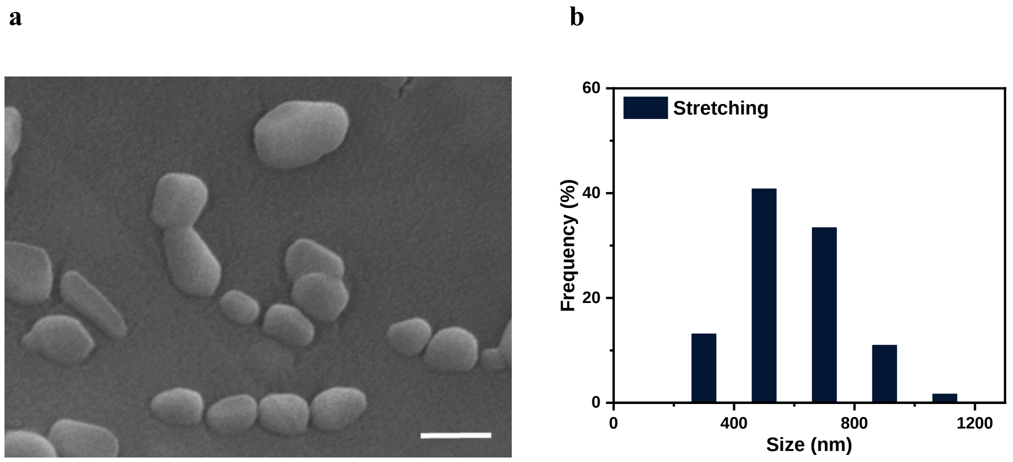

Supplementary Figure 14. a A typical SEM image of the SSH that was stretched to 300% its initial length. Scale bar, 500 nm. Each test was repeated three times independently with similar results. **b** Average diameter of the embedded liposomes in the same direction that the gel was stretched. The size was obtained through analyzing about 420 liposomes in the SEM images.

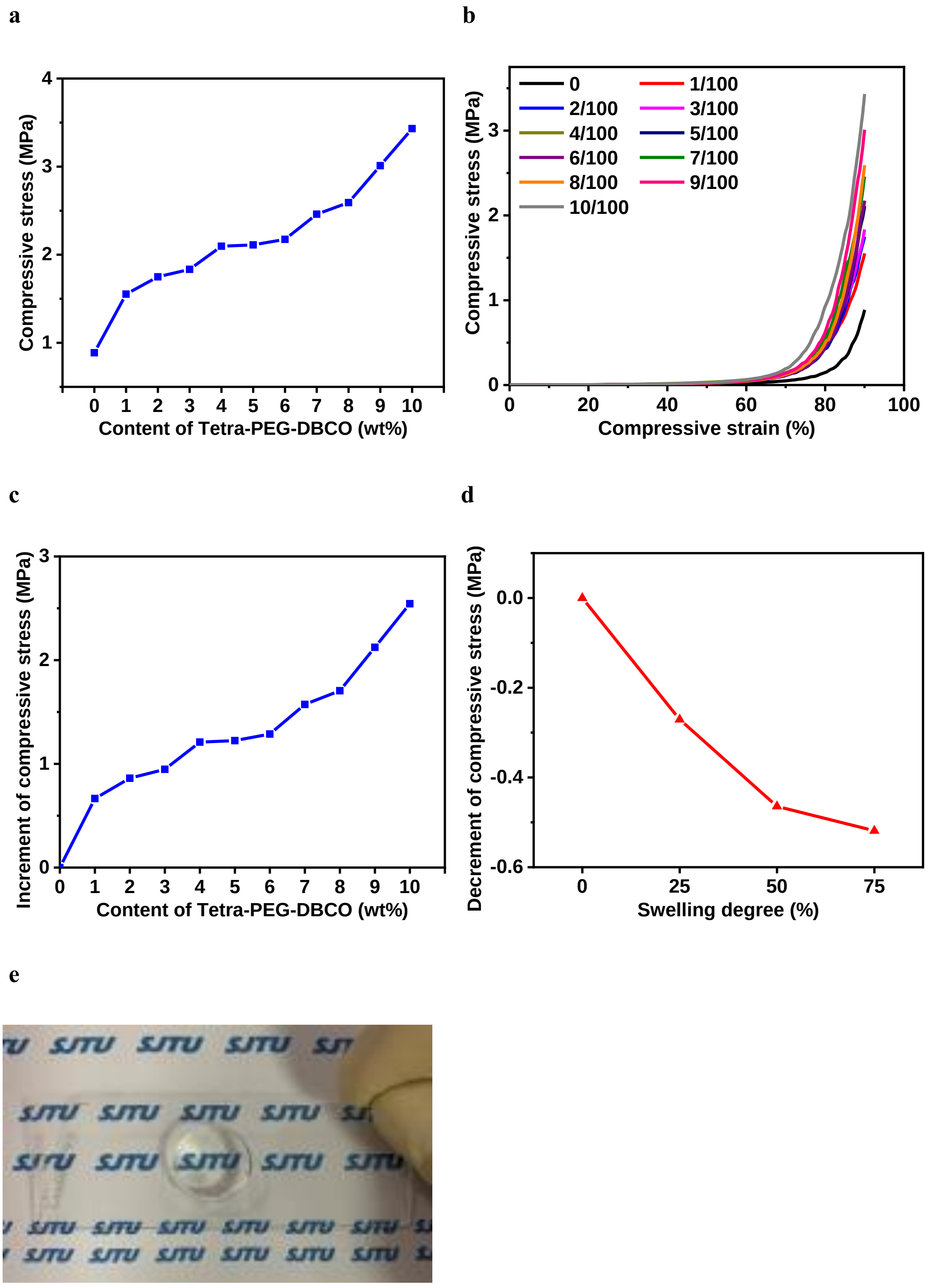

Supplementary Figure 15. a Variation of compressive stress, **b** compressive stress versus strain curves (ratios represent the weight percent of Tetra-PEG-DBCO) and **c** incremental variation of compressive stress under different loading of Tetra-PEG-DBCO. **d** Decrement of compressive stress of PAM with the increase of swelling degree. **e** A digital photo of the PAM/PEG double network hydrogel sample.

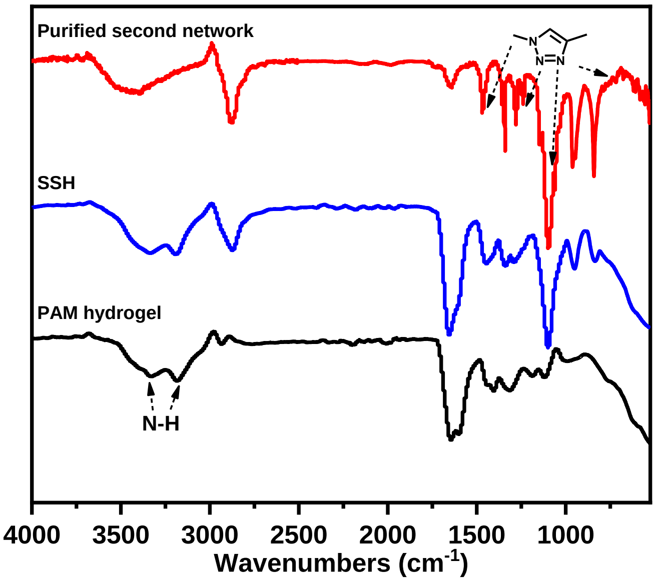

Supplementary Figure 16. FTIR spectra of PAM hydrogel, SSH and the purified second network.

Supplementary Table 1 Loading efficiency of N_3-PEG_3-N_3 in NALip under different molar feed ratios

Molar feed ratio[1]	0.5 : 1	1 : 1	2 : 1	3 : 1	4 : 1
Loading amount (mg)	0.67	1.13	2.36	2.83	2.73
Loading efficiency (%)	100	84.1	87.9	70.2	50.8
Ultrafiltration times	0	1	1	1	1

[1] Molar feed ratio: molar ratio of N_3-PEG_3-N_3 to HSPC during incubation
